# Supplementary material for: Multiple parallel origins of parasitic Marine Alveolates
Source: Nat Commun. 2023 Nov 3;14:7049. doi: 10.1038/s41467-023-42807-0 (PMC10624901; doi:10.1038/s41467-023-42807-0)
Supplement: Supplementary file 1 — Supplementary Information [file 41467_2023_42807_MOESM1_ESM.pdf]

Supplementary Information

Multiple parallel origins of parasitic Marine Alveolates

Corey C. Holt[1,2]†*, Elisabeth Hehenberger[1,3]†*, Denis V. Tikhonenkov[4,5], Victoria K. L. Jacko-Reynolds[1], Noriko Okamoto[1,2], Elizabeth C. Cooney[1,2], Nicholas A. T. Irwin[1,6], Patrick J. Keeling[1]*

[1]Department of Botany, University of British Columbia, Vancouver, British Columbia, Canada

[2]Hakai Institute, Heriot Bay, British Columbia, Canada

[3]Institute of Parasitology, Biology Centre Czech Academy of Sciences, České Budějovice, Czech Republic

[4]Papanin Institute for Biology of Inland Waters, Russian Academy of Sciences, Borok, Russia

[5]AquaBioSafe Laboratory, University of Tyumen, Tyumen, Russia

[6]Present address: Merton College, University of Oxford, Oxford, UK

†Equal contribution

*Corresponding authors: Corey C. Holt (corey.holt@ubc.ca), Elisabeth Hehenberger (elisabeth.hehenberger@paru.cas.cz), and Patrick J. Keeling (pkeeling@mail.ubc.ca)

Supplementary Figures

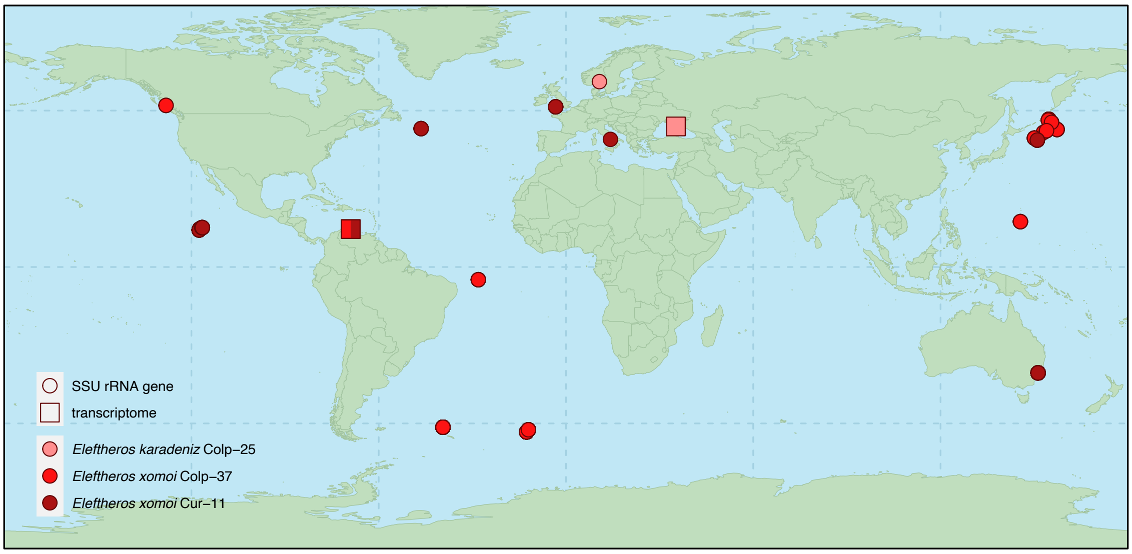

Supplementary Fig. 1: Global distribution of eleftherids. The global distribution of eleftherids within sequenced transcriptomes (squares) and environmental SSU rRNA amplicon studies (circles). Each point shows the presence of the corresponding strain (i.e., > 1 individual read) at those coordinates. All eleftherid reads were obtained from marine sediment with the exception of two SSU rRNA reads from the Coral Sea study PRJNA369575 (https://www.ncbi.nlm.nih.gov/bioproject/PRJNA369575/) which were discovered in the water column at 798m depth.

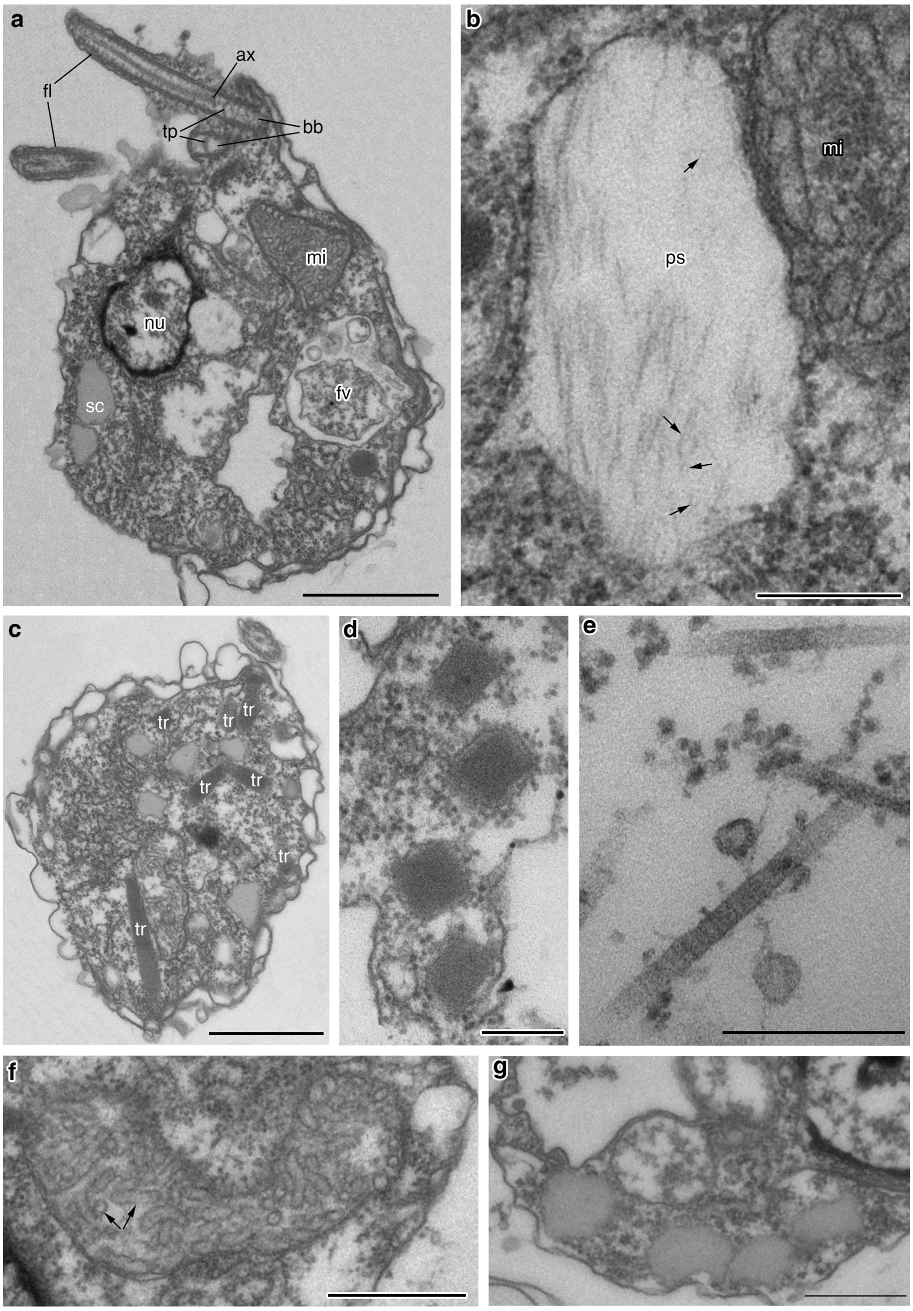

Supplementary Fig. 2 Cell structure of *Eleftheros karadeniz,* visualized by transmission electron microscopy. Related to Fig. 1. **a**, Longitudinal section showing arrangement of basal bodies and transitional zone of flagella with transverse plates. **b**, Perinuclear space with mastigonemes, tubular in cross-sections (arrows). **c**, Longitudinal sections and arrangement of trichocysts. **d**, Square cross-sections of trichocysts. **e**, Cross-striated filaments of discharged trichocysts. **f**, Mitochondrion with tubular cristae (arrows). **g**, Storage compounds. ax, axosome; bb, basal bodies; fl, flagella; fv, food vacuole; mi, mitochondrion; nu, nucleus; ps, perinuclear space; sc, storage compounds; tr, trichocyst; tp, transverse plate. Scale bars, 1

μm (**a, c**), 0,2 μm (**b, d, e**) and 0,5 μm (**f, g**). All observations were repeated at least 3 times with similar results.

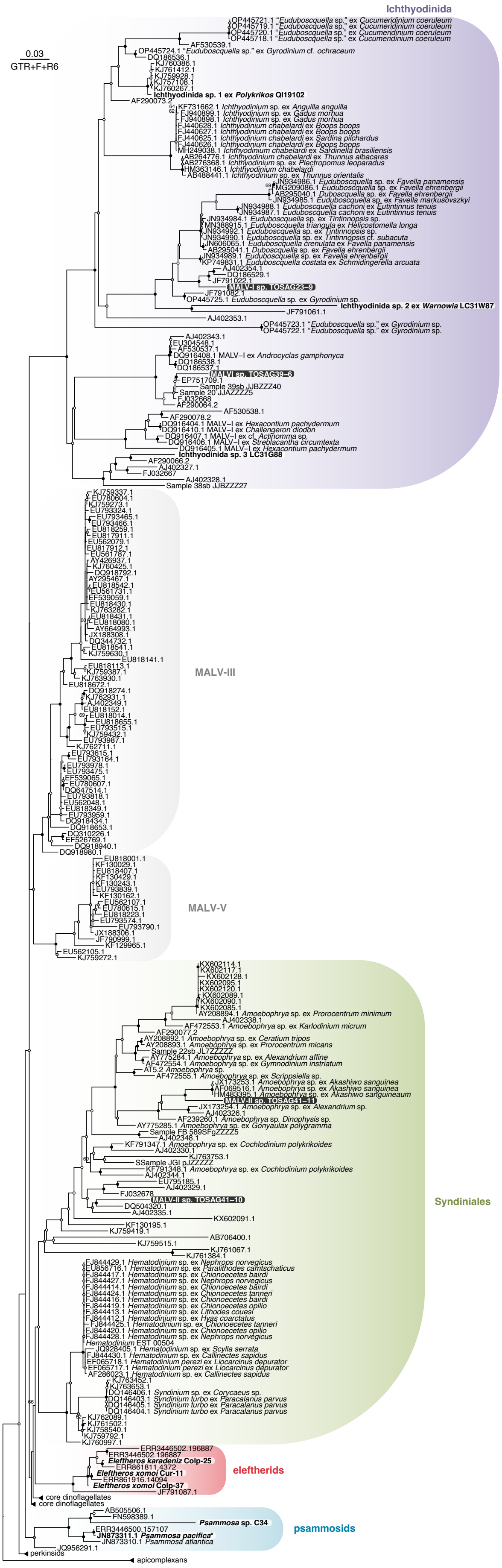
0.03
GTR+F+R6
Ichthyodinida
MALV-III
MALV-V
Syndiniales
eleftherids
psammosids
core dinoflagellates
perkinsids
apicomplexans

Supplementary Fig. 3 SSU rRNA gene phylogeny including short read sequences. ML analysis (GTR+F+R6) of MALV related sequences. Black dots at nodes represent full statistical support (UltraFast bootstrap = 100%). Grey dots represent 90% support and above (< 100). White dots represent 70% support and above (< 90). Support values are shown for support below 90%. Clades of interest coloured with proposed taxonomic groups: purple, Ichthyodinida (MALV-I); green, Syndiniales (MALV-II and MALV-IV); red, eleftherids; and blue, psammosids. Uncultured sequences are shown by accession numbers alone. Bold, black tip labels with white background show lineages isolated during this study, with the exception to JN873311.1 *Psammosa pacifica* (https://www.ncbi.nlm.nih.gov/nuccore/JN873311.1/) – which is 100% identical in sequence to *P. pacifica* Psp (a re-established culture from same location). Bold, white tip labels with black background show SAG datasets from Delmont *et al.* (2022). We were unable to retrieve an SSU rRNA gene sequence from MALV-I TOSAG41-9, MALV-II TOSAG47-5, MALV-II TOSAG47-7, AND MALV-II TOSAG48-3.

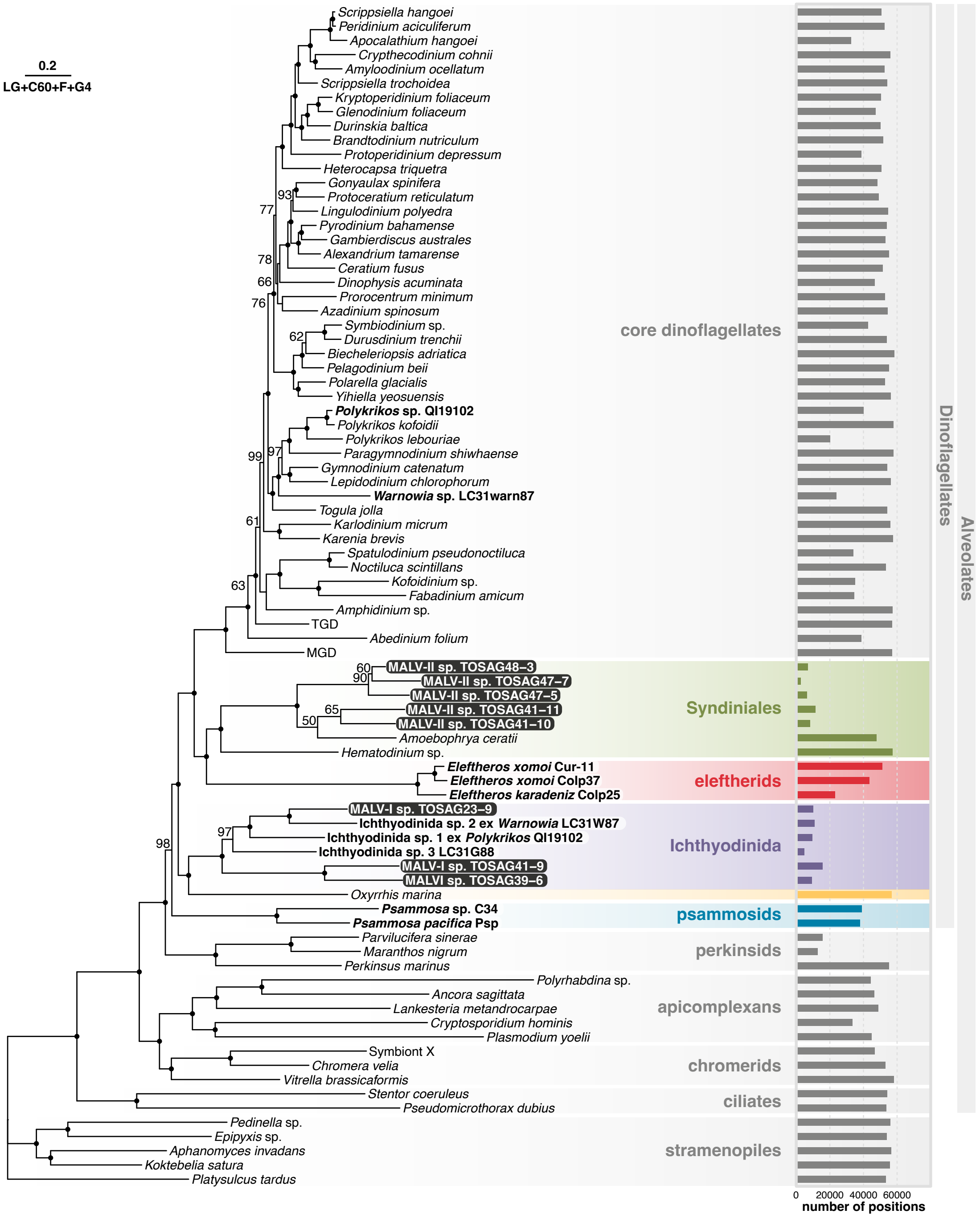

Supplementary Fig. 4 Phylogenomic reconstruction of the Marine Alveolates including SAG data. ML analysis of a 221 multi-protein alignment (LG+C60+F+G4) again separates MALV-I purple from the remaining Syndiniales (MALV-II and IV, green) to which the eleftherids (red) are the free-living sister group. Psammosids are shown in blue and *Oxyrrhis marina* in yellow. Black dots represent full statistical support (non-parametric bootstrap = 100%), values are shown for support below 100%. The percentage of amino acids present in

the corresponding alignment for each taxon are shown as bars to the right of the tree. Support for core dinoflagellate monophyly serves as a control for the presence of sufficient information for phylogenomic inference. Bold, black tip labels with white background show lineages isolated during this study. Bold, white tip labels with black background show SAG datasets from Delmont *et al.* (2022).

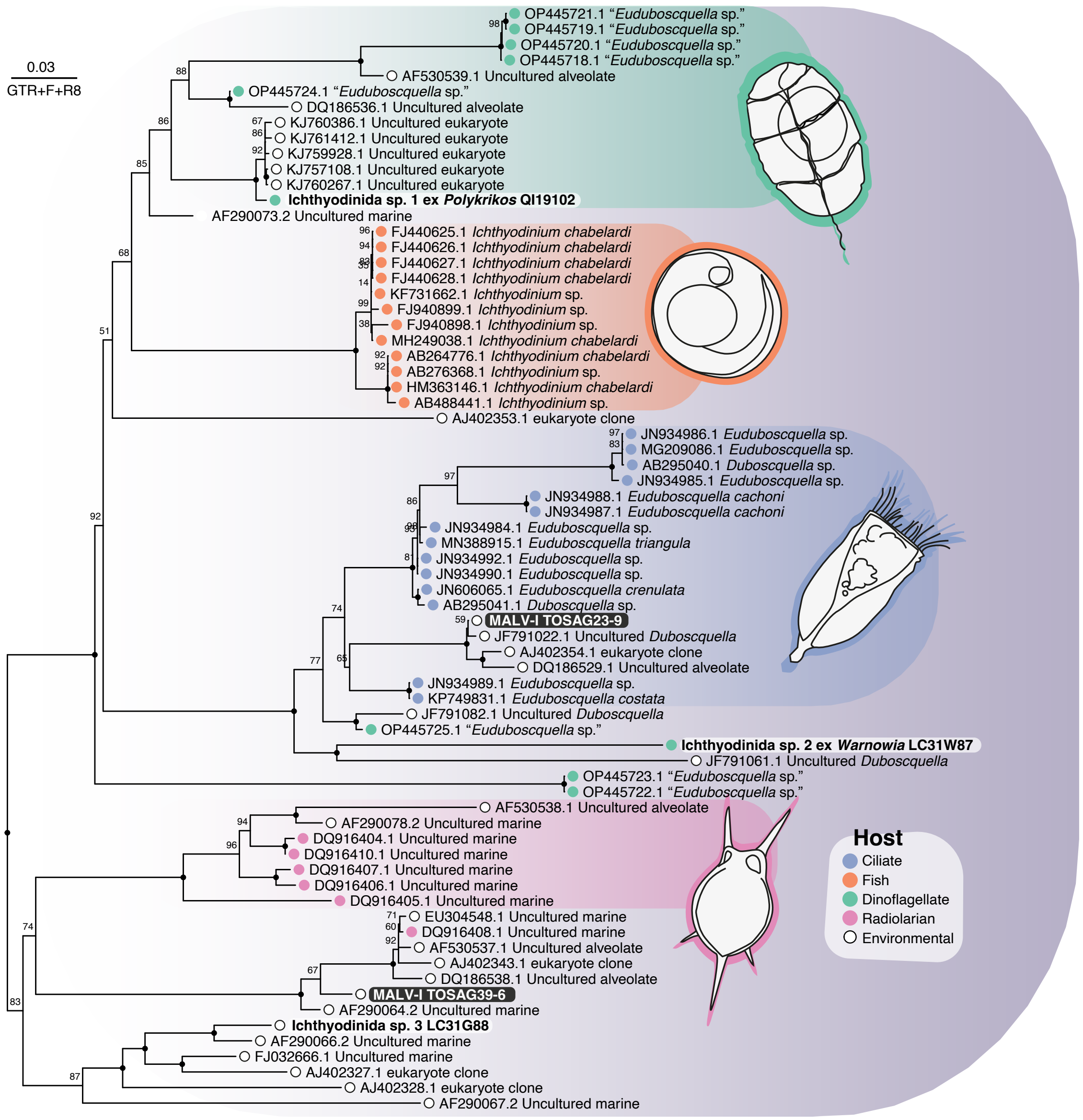

Supplementary Fig. 5 Phylogenetic reconstruction of rRNA SSU gene sequences including SAG data. ML analysis (GTR + F+ R8) showing MALV-I lineages isolated in this study with the addition of rRNA SSU gene sequences extracted from SAGs assembled in Delmont *et al.* (2022). Black dots at nodes represent full statistical support (UltraFast bootstrap = 100%), values are shown for support below 100%. Coloured circles accompanying MALV-I lineages reflect host identity: ciliates, blue; fish, orange; dinoflagellates, green; radiolarians, pink; and environmental, white. Bold, black tip labels

with white background show lineages isolated during this study. Bold, white tip labels with black background show SAG datasets from Delmont *et al.* (2022).

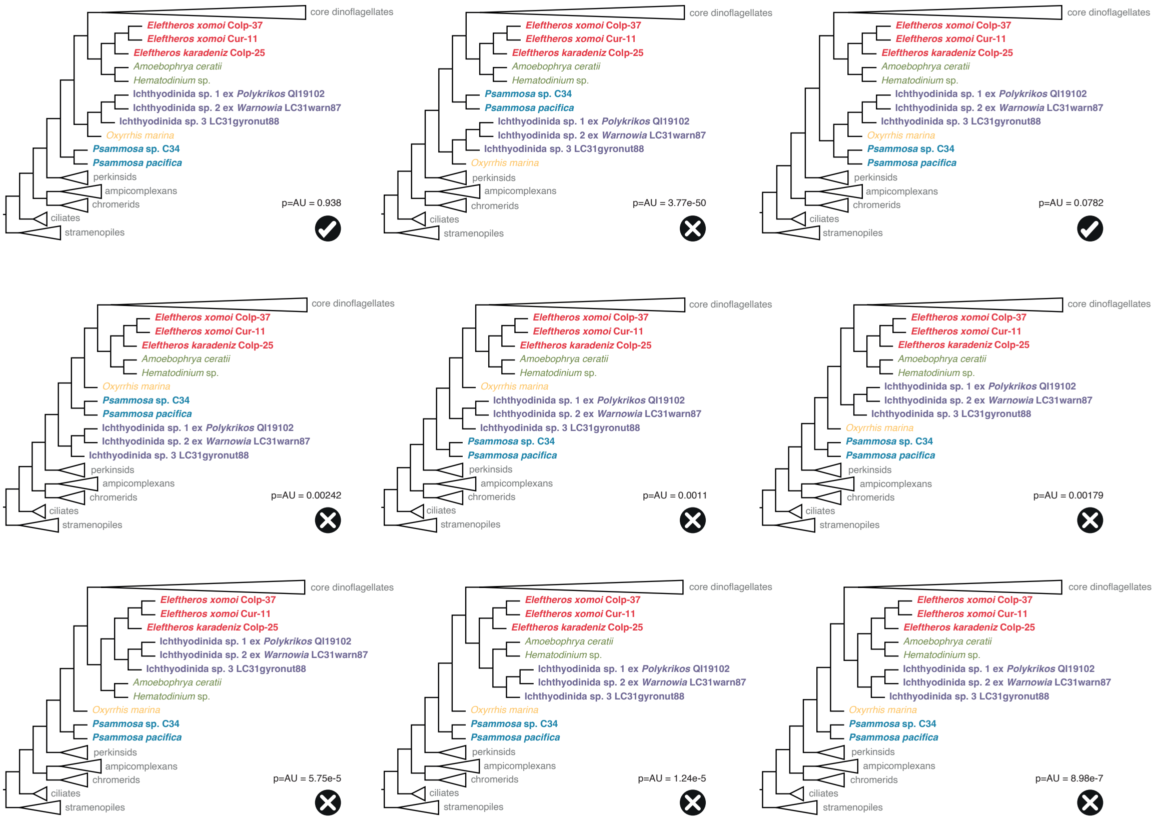

Supplementary Fig. 6 All topologies tested with approximately unbiased (AU) test. ML analysis of nine alternative topologies. Associated *p* values show two supported topologies (1 and 3) reflecting Fig. 2a and psammosids branching sister to *Oxyrrhis* and MALV-I. All topologies showing monophyly of MALVs (7,8,9) were rejected (p value < 0.05). Taxa coloured according to Fig. 2a: eleftherids, red; Syndiniales (MALV-II and -IV), green; Ichthyodinida (MALV-I), purple; *Oxyrrhis marina*, yellow; and psammosids, blue.

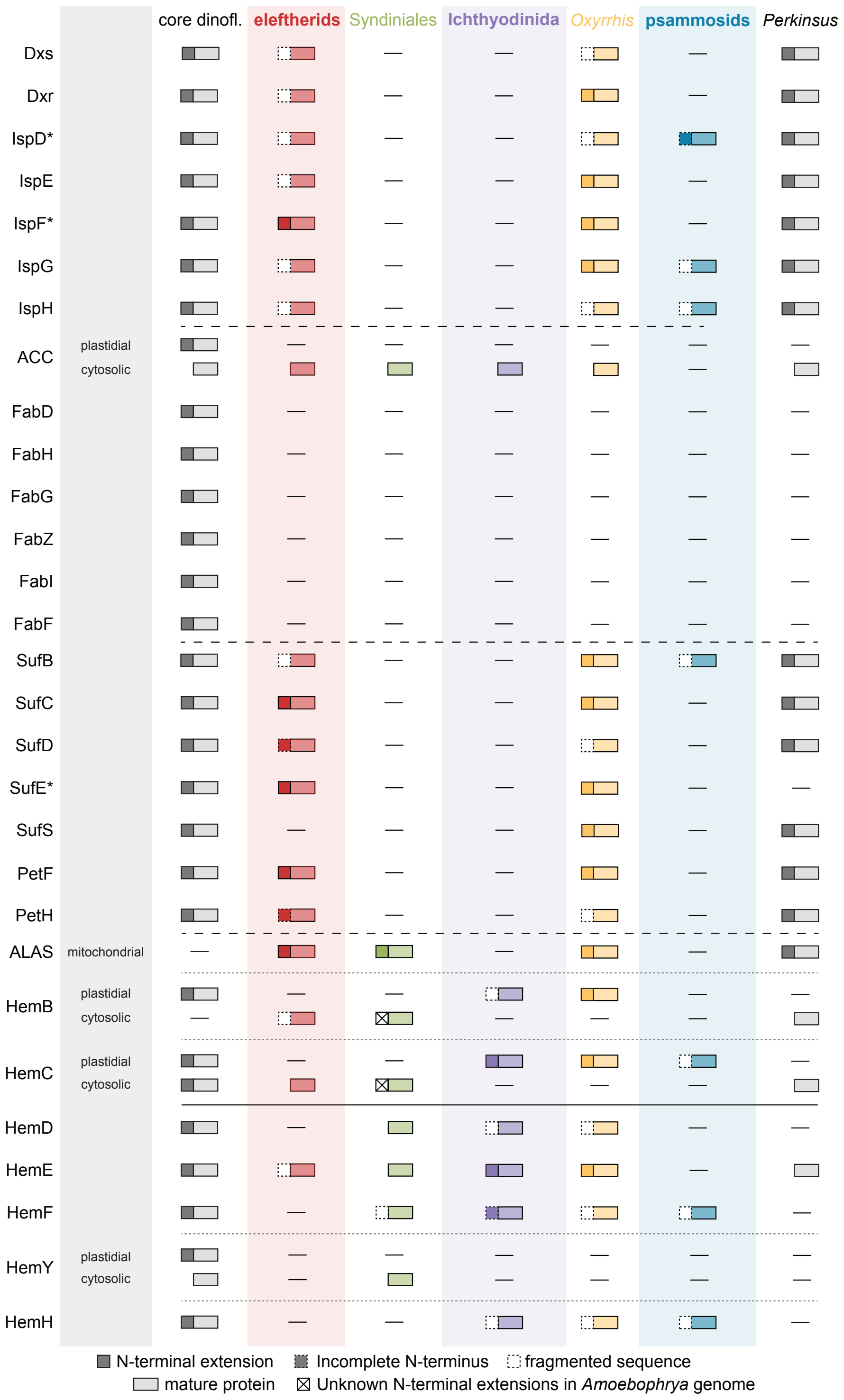

Supplementary Fig. 7: Plastid metabolic pathways in MALV-related lineages. Related to Fig. 3. Presence/absence table of plastid metabolic pathways in core dinoflagellates, eleftherids (red), Syndiniales (MALV-II and MALV-IV, green), Ichthyodinida (MALV-I, purple), *Oxyrrhis* (yellow), psammosids (blue) and *Perkinsus* including presence/absence of N-terminal extensions or incomplete N-termini. The pathways presented, from top to bottom,

are: isoprenoid, methylerythritol phosphate (MEP) pathway for isoprenoid biosynthesis; FASII, type 2 fatty acid biosynthesis pathway; FeS cluster, plastidial Fe-S cluster biosynthesis pathway including a ferrodoxin system; heme, heme biosynthesis. *, eleftherid sequences cluster with bacteria; m, mitochondrial; p/c, plastidial/cytosolic clade. HemH sequences from *Oxyrrhis* are highly fragmented and not included in the corresponding phylogeny.

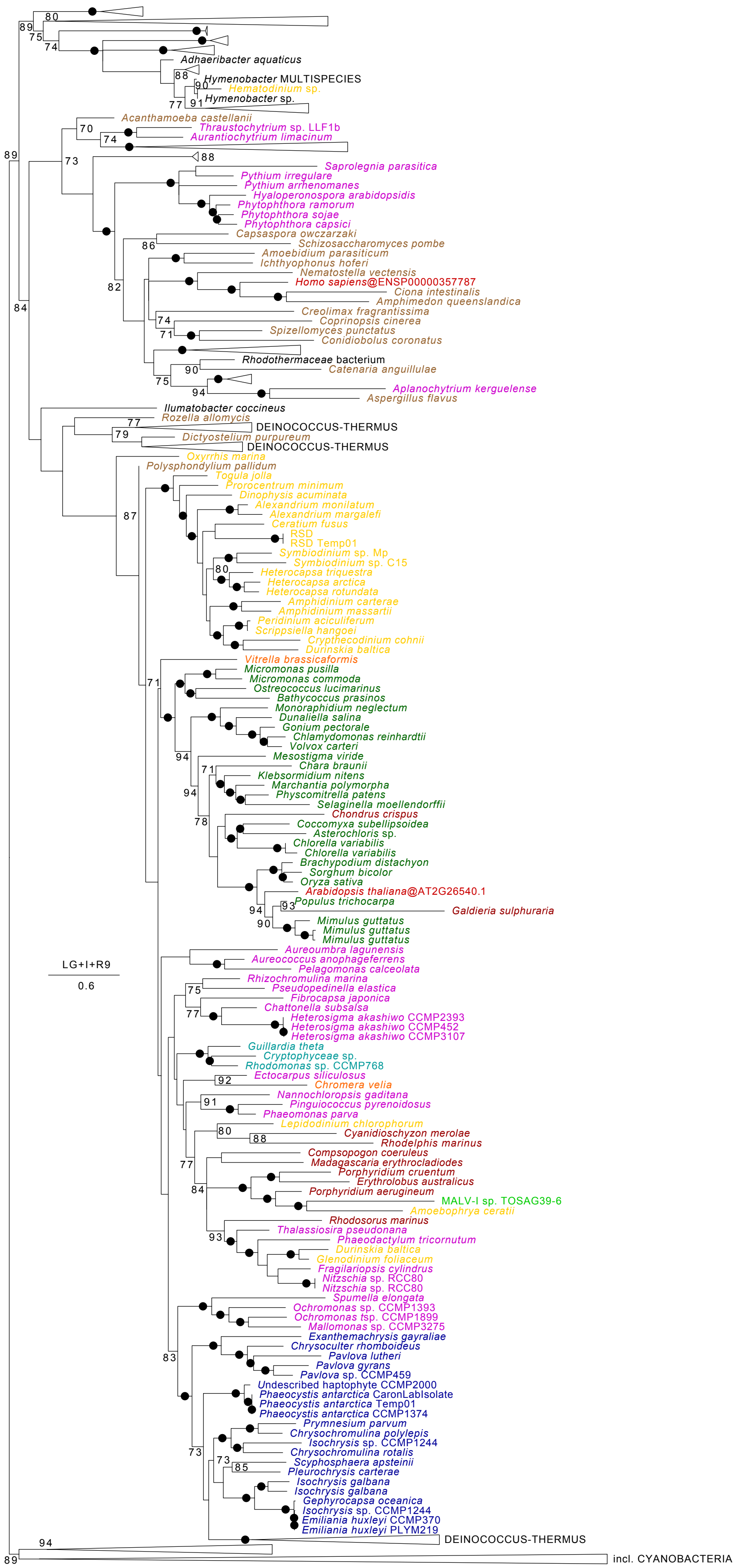
Adhaeribacter aquaticus
Hymenobacter MULTISPECIES
Hematodinium sp.
Hymenobacter sp.
Acanthamoeba castellanii
Thraustochytrium sp. LLF1b
Aurantiochytrium limacinum
Saprolegnia parasitica
Pythium irregulare
Pythium arrhenomanes
Hyaloperonospora arabidopsidis
Phytophthora ramorum
Phytophthora sojae
Phytophthora capsici
Capsaspora owczarzaki
Schizosaccharomyces pombe
Amoebidium parasiticum
Ichthyophonus hoferi
Nematostella vectensis
Homo sapiens@ENSP00000357787
Ciona intestinalis
Amphimedon queenslandica
Creolimax fragrantissima
Coprinopsis cinerea
Spizellomyces punctatus
Conidiobolus coronatus
Rhodothermaceae bacterium
Catenaria anguillulae
Aplanochytrium kerguelense
Aspergillus flavus
Ilumatobacter coccineus
Rozella allomycis
DEINOCOCCUS-THERMUS
Dictyostelium purpureum
DEINOCOCCUS-THERMUS
Oxyrrhis marina
Polysphondylium pallidum
Togula jolla
Prorocentrum minimum
Dinophysis acuminata
Alexandrium monilatum
Alexandrium margalefi
Ceratium fusus
RSD
RSD Temp01
Symbiodinium sp. Mp
Symbiodinium sp. C15
Heterocapsa triquestra
Heterocapsa arctica
Heterocapsa rotundata
Amphidinium carterae
Amphidinium massartii
Peridinium aciculiferum
Scrippsiella hangoei
Crypthecodinium cohnii
Durinskia baltica
Vitrella brassicaformis
Micromonas pusilla
Micromonas commoda
Ostreococcus lucimarinus
Bathycoccus prasinos
Monoraphidium neglectum
Dunaliella salina
Gonium pectorale
Chlamydomonas reinhardtii
Volvox carteri
Mesostigma viride
Chara braunii
Klebsormidium nitens
Marchantia polymorpha
Physcomitrella patens
Selaginella moellendorffii
Chondrus crispus
Coccomyxa subellipsoidea
Asterochloris sp.
Chlorella variabilis
Chlorella variabilis
Brachypodium distachyon
Sorghum bicolor
Oryza sativa
Arabidopsis thaliana@AT2G26540.1
Populus trichocarpa
Galdieria sulphuraria
Mimulus guttatus
Mimulus guttatus
Mimulus guttatus
LG+I+R9
0.6
Aureoumbra lagunensis
Aureococcus anophagefferens
Pelagomonas calceolata
Rhizochromulina marina
Pseudopedinella elastica
Fibrocapsa japonica
Chattonella subsalsa
Heterosigma akashiwo CCMP2393
Heterosigma akashiwo CCMP452
Heterosigma akashiwo CCMP3107
Guillardia theta
Cryptophyceae sp.
Rhodomonas sp. CCMP768
Ectocarpus siliculosus
Chromera velia
Nannochloropsis gaditana
Pinguiococcus pyrenoidosus
Phaeomonas parva
Lepidodinium chlorophorum
Cyanidioschyzon merolae
Rhodelphis marinus
Compsopogon coeruleus
Madagascaria erythrocladiodes
Porphyridium cruentum
Erythrolobus australicus
Porphyridium aerugineum
MALV-I sp. TOSAG39-6
Amoebophrya ceratii
Rhodosorus marinus
Thalassiosira pseudonana
Phaeodactylum tricornutum
Durinskia baltica
Glenodinium foliaceum
Fragilariopsis cylindrus
Nitzschia sp. RCC80
Nitzschia sp. RCC80
Spumella elongata
Ochromonas sp. CCMP1393
Ochromonas tsp. CCMP1899
Mallomonas sp. CCMP3275
Exanthemachrysis gayraliae
Chrysoculter rhomboideus
Pavlova lutheri
Pavlova gyrans
Pavlova sp. CCMP459
Undescribed haptophyte CCMP2000
Phaeocystis antarctica CaronLabIsolate
Phaeocystis antarctica Temp01
Phaeocystis antarctica CCMP1374
Prymnesium parvum
Chrysochromulina polylepis
Isochrysis sp. CCMP1244
Chrysochromulina rotalis
Scyphosphaera apsteinii
Pleurochrysis carterae
Isochrysis galbana
Isochrysis galbana
Gephyrocapsa oceanica
Isochrysis sp. CCMP1244
Emiliania huxleyi CCMP370
Emiliania huxleyi PLYM219
DEINOCOCCUS-THERMUS
incl. CYANOBACTERIA

Supplementary Fig. 8: Maximum-likelihood phylogeny of HemD. The scale bar and the number beneath it indicate the estimated number of substitutions per site, above the scale bar the model for tree reconstruction is indicated. Node numbers represent ultrafast bootstrap support values of >70%, black dots indicate support values of >= 95%. Eukaryotic groups are indicated by colored taxon names: green, Viridiplantae; dark red, rhodophytes; grey, glaucophytes; turquoise, cryptophytes; pink, stramenopiles; dark blue, haptophytes; yellow, Dinozoa; light green, MALV-I. Black taxa/clades outlined in black are prokaryotic. Annotated orthologs in model species are indicated by red taxon name and protein identifier. For species represented by more than one strain or taxa identified on genus level only, the strain information is provided where available.

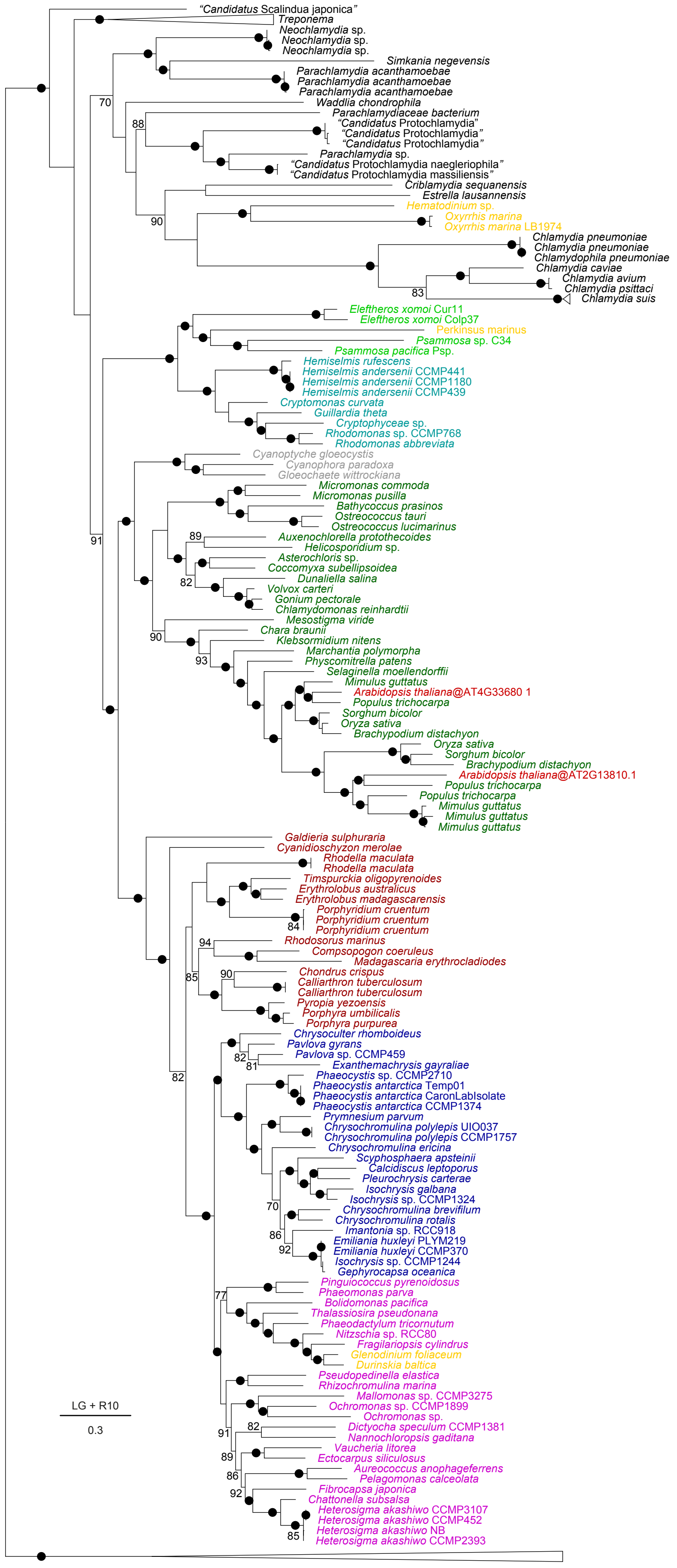
"Candidatus Scalindua japonica"
Treponema
Neochlamydia sp.
Neochlamydia sp.
Neochlamydia sp.
Simkania negevensis
Parachlamydia acanthamoebae
Parachlamydia acanthamoebae
Parachlamydia acanthamoebae
Waddlia chondrophila
Parachlamydiaceae bacterium
"Candidatus Protochlamydia"
"Candidatus Protochlamydia"
"Candidatus Protochlamydia"
Parachlamydia sp.
"Candidatus Protochlamydia naegleriophila"
"Candidatus Protochlamydia massiliensis"
Criblamydia sequanensis
Estrella lausannensis
Hematodinium sp.
Oxyrrhis marina
Oxyrrhis marina LB1974
Chlamydia pneumoniae
Chlamydia pneumoniae
Chlamydophila pneumoniae
Chlamydia caviae
Chlamydia avium
Chlamydia psittaci
Chlamydia suis
Eleftheros xomoi Cur11
Eleftheros xomoi Colp37
Perkinsus marinus
Psammosa sp. C34
Psammosa pacifica Psp.
Hemiselmis rufescens
Hemiselmis andersenii CCMP441
Hemiselmis andersenii CCMP1180
Hemiselmis andersenii CCMP439
Cryptomonas curvata
Guillardia theta
Cryptophyceae sp.
Rhodomonas sp. CCMP768
Rhodomonas abbreviata
Cyanoptyche gloeocystis
Cyanophora paradoxa
Gloeochaete wittrockiana
Micromonas commoda
Micromonas pusilla
Bathycoccus prasinos
Ostreococcus tauri
Ostreococcus lucimarinus
Auxenochlorella protothecoides
Helicosporidium sp.
Asterochloris sp.
Coccomyxa subellipsoidea
Dunaliella salina
Volvox carteri
Gonium pectorale
Chlamydomonas reinhardtii
Mesostigma viride
Chara braunii
Klebsormidium nitens
Marchantia polymorpha
Physcomitrella patens
Selaginella moellendorffii
Mimulus guttatus
Arabidopsis thaliana@AT4G33680 1
Populus trichocarpa
Sorghum bicolor
Oryza sativa
Brachypodium distachyon
Oryza sativa
Sorghum bicolor
Brachypodium distachyon
Arabidopsis thaliana@AT2G13810.1
Populus trichocarpa
Populus trichocarpa
Mimulus guttatus
Mimulus guttatus
Mimulus guttatus
Galdieria sulphuraria
Cyanidioschyzon merolae
Rhodella maculata
Rhodella maculata
Timspurckia oligopyrenoides
Erythrolobus australicus
Erythrolobus madagascarensis
Porphyridium cruentum
Porphyridium cruentum
Porphyridium cruentum
Rhodosorus marinus
Compsopogon coeruleus
Madagascaria erythrocladiodes
Chondrus crispus
Calliarthron tuberculosum
Calliarthron tuberculosum
Pyropia yezoensis
Porphyra umbilicalis
Porphyra purpurea
Chrysoculter rhomboideus
Pavlova gyrans
Pavlova sp. CCMP459
Exanthemachrysis gayraliae
Phaeocystis sp. CCMP2710
Phaeocystis antarctica Temp01
Phaeocystis antarctica CaronLabIsolate
Phaeocystis antarctica CCMP1374
Prymnesium parvum
Chrysochromulina polylepis UIO037
Chrysochromulina polylepis CCMP1757
Chrysochromulina ericina
Scyphosphaera apsteinii
Calcidiscus leptoporus
Pleurochrysis carterae
Isochrysis galbana
Isochrysis sp. CCMP1324
Chrysochromulina brevifilum
Chrysochromulina rotalis
Imantonia sp. RCC918
Emiliania huxleyi PLYM219
Emiliania huxleyi CCMP370
Isochrysis sp. CCMP1244
Gephyrocapsa oceanica
Pinguiococcus pyrenoidosus
Phaeomonas parva
Bolidomonas pacifica
Thalassiosira pseudonana
Phaeodactylum tricornutum
Nitzschia sp. RCC80
Fragilariopsis cylindrus
Glenodinium foliaceum
Durinskia baltica
Pseudopedinella elastica
Rhizochromulina marina
Mallomonas sp. CCMP3275
Ochromonas sp. CCMP1899
Ochromonas sp.
Dictyocha speculum CCMP1381
Nannochloropsis gaditana
Vaucheria litorea
Ectocarpus siliculosus
Aureococcus anophageferrens
Pelagomonas calceolata
Fibrocapsa japonica
Chattonella subsalsa
Heterosigma akashiwo CCMP3107
Heterosigma akashiwo CCMP452
Heterosigma akashiwo NB
Heterosigma akashiwo CCMP2393
LG + R10
0.3

Supplementary Fig. 9: Maximum-likelihood phylogeny of DapL. The scale bar and the number beneath it indicate the estimated number of substitutions per site, above the scale bar the model for tree reconstruction is indicated. Node numbers represent ultrafast bootstrap support values of >70%, black dots indicate support values of >= 95%. Eukaryotic groups are indicated by colored taxon names: green, Viridiplantae; dark red, rhodophytes; grey, glaucophytes; turquoise, cryptophytes; pink, stramenopiles; dark blue, haptophytes; yellow, Dinozoa; light green, eleftherids. Black taxa/clades outlined in black are prokaryotic. Annotated orthologs in model species are indicated by red taxon name and protein identifier. For species represented by more than one strain or taxa identified on genus level only, the strain information is provided where available.
