# Supplementary material for: Multiple parallel origins of parasitic Marine Alveolates
Source: Nat Commun. 2023 Nov 3;14:7049. doi: 10.1038/s41467-023-42807-0 (PMC10624901; doi:10.1038/s41467-023-42807-0)
Supplement: Supplementary file 3 — Description of Additional Supplementary Files [file 41467_2023_42807_MOESM3_ESM.pdf]

Description of Additional Supplementary Files

File Name: Supplementary Data 1
Description: Amplicon studies seared for environmental distribution and abundance. Tab 1, amplicons over 97 % identity to eleftherid/psammosid SSU rRNA gene sequences. Tab 2, metadata describing 27 studies used for search.

File Name: Supplementary Data 2
Description: Putative plastid-targeted proteins in eleftherids (Colp-37, Cur-11 and Colp-25), MALV-I (TOSAG39-6 and TOSAG41-9 from Delmont et al. 2022), and psammosids (P.pacifica and Psammosa sp. C34). Coloured segments within sequences indicate transmembrane regions within N-terminal extensions).

File Name: Supplementary Movie 1
Description: Swimming cells of *Eleftheros xomoi* and flagella beating, visualised by light microscopy.

File Name: Supplementary Movie 2
Description: Live cell of Ichthyodinida sp.1 ex *Polykrikos* sp. (MALV-I), visualised by light microscopy.

File Name: Supplementary Movie 3
Description: Live cell of Ichthyodinida sp.2 ex *Warnowia* sp. (MALV-I), visualised by light microscopy.

File Name: Supplementary Movie 4
Description: Live cell of Ichthyodinida sp.3 (MALV-I), visualised by light microscopy.
