# Supplementary material for: Multiple parallel origins of parasitic Marine Alveolates
Source: Nat Commun. 2023 Nov 3;14:7049. doi: 10.1038/s41467-023-42807-0 (PMC10624901; doi:10.1038/s41467-023-42807-0)
Supplement: Supplementary file 10 — Reporting Summary [file 41467_2023_42807_MOESM10_ESM.pdf]

Corresponding author(s): Corey C. Holt, Elisabeth Hehenberger, Patrick J. Keeling

Last updated by author(s): Oct 12, 2023

Reporting Summary

Nature Portfolio wishes to improve the reproducibility of the work that we publish. This form provides structure for consistency and transparency in reporting. For further information on Nature Portfolio policies, see our Editorial Policies and the Editorial Policy Checklist.

Statistics

For all statistical analyses, confirm that the following items are present in the figure legend, table legend, main text, or Methods section.

n/a	Confirmed	
[ ]	[x]	The exact sample size (n) for each experimental group/condition, given as a discrete number and unit of measurement
[x]	[ ]	A statement on whether measurements were taken from distinct samples or whether the same sample was measured repeatedly
[ ]	[x]	The statistical test(s) used AND whether they are one- or two-sided<br>*Only common tests should be described solely by name; describe more complex techniques in the Methods section.*
[x]	[ ]	A description of all covariates tested
[x]	[ ]	A description of any assumptions or corrections, such as tests of normality and adjustment for multiple comparisons
[x]	[ ]	A full description of the statistical parameters including central tendency (e.g. means) or other basic estimates (e.g. regression coefficient) AND variation (e.g. standard deviation) or associated estimates of uncertainty (e.g. confidence intervals)
[ ]	[x]	For null hypothesis testing, the test statistic (e.g. F, t, r) with confidence intervals, effect sizes, degrees of freedom and P value noted<br>*Give P values as exact values whenever suitable.*
[x]	[ ]	For Bayesian analysis, information on the choice of priors and Markov chain Monte Carlo settings
[x]	[ ]	For hierarchical and complex designs, identification of the appropriate level for tests and full reporting of outcomes
[x]	[ ]	Estimates of effect sizes (e.g. Cohen's d, Pearson's r), indicating how they were calculated

Our web collection on statistics for biologists contains articles on many of the points above.

Software and code

Policy information about availability of computer code

Data collection	Sequence quality: FastQC v0.10.1; Cutadapt v.3.2<br>Transcriptome assembly: PEAR v0.9.6; Trinity v2.0.6; rnaSPAdes v.3.15.1;TransDecoder v5.0.2
Data analysis	Transcriptome analysis: BLAST+ v2.2.30; CD-HIT v4.6; HMMER3.1 (hmmer.org)<br><br>Phylogenomic and phylogenetic analysis: MAFFT v7.222; trimAL v1.2; FastTree v2.1.7; RAxML v8.1.6; SCaFOs v1.2.5; IQ-TREE v1.6.5; PREQUAL v1.02; Divvier v1.01; R v.4.1.2; ggtree_3.2.1; treeio_1.18.1<br><br>Environmental analysis: sratoolkit v2.10.8; MICCA v1.7.2; QIIME2 v.2020.11.1 ; R v.4.1.2; rnaturalearth_0.3.2

For manuscripts utilizing custom algorithms or software that are central to the research but not yet described in published literature, software must be made available to editors and reviewers. We strongly encourage code deposition in a community repository (e.g. GitHub). See the Nature Portfolio guidelines for submitting code & software for further information.

Data

Policy information about availability of data

All manuscripts must include a data availability statement. This statement should provide the following information, where applicable:

- Accession codes, unique identifiers, or web links for publicly available datasets
- A description of any restrictions on data availability
- For clinical datasets or third party data, please ensure that the statement adheres to our policy

Raw transcriptome reads have been deposited in the GenBank Sequence Read Archive (SRA) database under the accessions SRR25604407 - SRR25604420. SSU

rRNA gene sequences retrieved from the transcriptomes have also been deposited in GenBank under the accessions OR427349-OR427355. All sequence data is linked to the BioProject accession code PRJNA1003956 (https://www.ncbi.nlm.nih.gov/bioproject/PRJNA1003956). Assembled transcriptomes, along with individual gene alignments, concatenated and trimmed alignments, and ML and Bayesian tree files for the phylogenomic dataset are available at Figshare (https://figshare.com/s/fd3170a4b4a05027d9d2). The untrimmed and trimmed alignments, alignments depicting N-terminal extensions and tree files in nexus and pdf format for plastid-associated and other proteins of interest are available at Figshare (https://figshare.com/s/fd3170a4b4a05027d9d2). The genus Eleftheros (urn:lsid:zoobank.org:act:DD5CBE62-6058-4ADA-BDBD-7B3A486447E1) and species Eleftheros xomoi (urn:lsid:zoobank.org:act:1B42392D-9FE1-464F-B637-50761EFC7A08) and Eleftheros karadeniz (urn:lsid:zoobank.org:act:6264A9FC-BBD1-4D8D-B0E6-EB05C540D628) have been registered with the Zoobank database (http://zoobank.org/). Zoobank Registration: LSID for this publication: urn:lsid:zoobank.org:pub:CBA7B765-7996-4F42-8AAF-42460CE7F77E

Field-specific reporting

Please select the one below that is the best fit for your research. If you are not sure, read the appropriate sections before making your selection.

☐ Life sciences ☐ Behavioural & social sciences ☒ Ecological, evolutionary & environmental sciences

For a reference copy of the document with all sections, see nature.com/documents/nr-reporting-summary-flat.pdf

Ecological, evolutionary & environmental sciences study design

All studies must disclose on these points even when the disclosure is negative.

Study description	In this study, we show that the Marine Alveolates are polyphyletic and parasitism evolved twice before the divergence of the core dinoflagellates. Transcriptomic data from several new taxa shows that free-living eleftherids are the sister group to MALV II/IV while MALV I is the sister lineage to Oxyrrhis marina. Furthermore, MALV-I data show clear evidence of plastid-targeted proteins involved in Heme biosynthesis. Our data ultimately show MALV II/IV and MALV I arose independently from two distinct, free-living heterotrophs.
Research sample	This research describes two new species, Eleftheros xomoi and E. karadeniz from a new genus of predatory eukaryotic microbes that are the closest known relatives to MALV II/IV. The organisms were collected from the surface of corals in coastal waters and near shore seawater sediments, respectively. We also add three MALV I lineages isolated from infected dinoflagellates (Polykrikos sp., Warnowia sp., and an unknown host) collected during a plankton tow. Finally, we include data from two Psammosa species, previously isolated from marine sediment.
Sampling strategy	Sample size is not relevant to the present study.
Data collection	Samples were collected from coral surface, marine sediments and water column, and the new organisms were subsequently grown in the laboratory. Microscopic data were recorded by D Tikhonenkov, C Holt and E Cooney. Sequencing data were generated by The Centre for Applied Genomics, Next Generation Sequencing Facility, SickKids (Eleftheros xomoi strain 2 (Cur-11)) and Sequencing and Bioinformatic Consortium, The University of British Columbia (E. karadeniz (Colp-25), E. xomoi strain 1 (Colp-37), Ichthyodinida sp. 1, Ichthyodinida sp. 2, Ichthyodinida sp. 3, Psammosa pacifica (Psp) and Psammosa sp. (C34)). Transcriptome data were assembled by C Holt, E Hehenberger, V Jacko-Reynolds and E Cooney. Single-cell Amplified Genomes (SAGs) from Delmont et al. (2022) were also added.
Timing and spatial scale	Sampling was carried out from the surface of marine corals in coastal waters of Curacao in April 2016 and April 2018; from near-shore sediment samples in the Black Sea, near Crimea, in May 2015 and 2016; from near-shore sediment samples and plankton net tows in British Columbia in 2010 and 2022, respectively; and near-shore plankton net tows in Curacao in 2023. We had no reason to expect to find the organisms that we did, so there is no specific rationale to sampling sites.
Data exclusions	Sequencing data from prey organisms were excluded from the analyses for the eleftherids (Colp-25 and Colp37) and psammosids (Psp and C34). To do this, we subtracted transcripts derived from prey and any non-eukaryotic transcripts from the total datasets. Host data was excluded from the Deorella dataset during phylogenetic analyses. The raw data associated with this will be accessible in the raw read files deposited in the NCBI SRA database.
Reproducibility	Microscopic analyses were conducted several times. Phylogenomic analyses were carried out with a number of different approaches (Maximum Likelihood, fast-evolving site removal) and all associated datasets will be made available.
Randomization	Randomization is not relevant to the present study because organisms were not allocated into groups.
Blinding	Blinding was not relevant to the present study.

Did the study involve field work? ☒ Yes ☐ No

Field work, collection and transport

Field conditions	Climatic conditions in the field were not recorded and are not relevant to the study.
Location	1) Carribean Sea near Curacao (12°06'34.3"N, 68°57'15.4"W and 12°07'28.6"N 68°58'06.4"W)<br>2) Black Sea near T.I. Vyazemsky Karadag Scientific Station, Crimea (44°54'40.8" N; 35°12'2.3" E)<br>3) Heriot Bay, Quadra Island, British Columbia, Canada (50°06'10.6"N 125°12'46.5"W)<br>4) Boundary Bay, British Columbia, Canada (49.0086 N 123.0228 W)

Access & import/export	Habitats were accessed via a car and scuba diving gear and a car. No permissions were required for sampling in the selected sampling sites.
Disturbance	No disturbances to the sites were caused.

Reporting for specific materials, systems and methods

We require information from authors about some types of materials, experimental systems and methods used in many studies. Here, indicate whether each material, system or method listed is relevant to your study. If you are not sure if a list item applies to your research, read the appropriate section before selecting a response.

Materials & experimental systems

n/a	Involved in the study
☒	☐ Antibodies
☒	☐ Eukaryotic cell lines
☒	☐ Palaeontology and archaeology
☐	☒ Animals and other organisms
☒	☐ Human research participants
☒	☐ Clinical data
☒	☐ Dual use research of concern

Methods

n/a	Involved in the study
☒	☐ ChIP-seq
☒	☐ Flow cytometry
☒	☐ MRI-based neuroimaging

Animals and other organisms

Policy information about studies involving animals; ARRIVE guidelines recommended for reporting animal research

Laboratory animals	The study did not involve laboratory animals.
Wild animals	The study did not involve wild animals.
Field-collected samples	Monoeukaryotic cultures of Eleftheros xomoi and E. karadeniz were established by isolating cells with a glass micropipette. Cultures were maintained at 22 °C. E xomoi and E. karadeniz were propagateed using the kinetoplastid protist Procryptobia sorokini B-69 as prey. The kinetoplastid was grown in marine Schmalz-Pratt's medium and preyed upon Pseudomonas fluorescens.<br>Psammosa was cultured in a modified K-Si medium at 18 °C with the bacterivorous stramenopile (Tofino-D6Ga) as a food source (Okamoto et al 2012).
Ethics oversight	No ethical approval was required. The organisms described here are novel eukaryotic microbes (protists) that feed on other protists and pose no risk.

Note that full information on the approval of the study protocol must also be provided in the manuscript.
